# Barriers in Access to the Treatment for People with Gambling Disorders. Are They Different from Those Experienced by People with Alcohol and/or Drug Dependence?

**DOI:** 10.1007/s10899-016-9655-1

**Published:** 2016-11-10

**Authors:** Katarzyna Dąbrowska, Jacek Moskalewicz, Łukasz Wieczorek

**Affiliations:** Department of Studies on Alcoholism and Drug Dependence, Institute of Psychiatry and Neurology, Sobieskiego 9 Street, 02-957 Warsaw, Poland

**Keywords:** Gambling disorders, Treatment, Barriers, Alcohol disorders, Drug disorders

## Abstract

A prevalence of gambling disorders is diversified depending on the region of the world. Almost three quarters of pathological gamblers had never sought a professional treatment as well as an assistance in self-help groups. Reasons why they do not initiate a treatment are complex. The aim of the article is to compare barriers to the treatment for people with gambling disorders found in presented study and barriers to alcohol and drug treatment identified in the available literature. The semi structured interviews were applied and conducted with people with gambling disorders, social workers, therapists employed in the addiction treatment facilities, General Practitioners and psychiatrists. Selection of the respondents was based on purposive sampling. In total, 90 interviews were completed. Respondents identified individual barriers as well as structural ones. Individual barriers include internal resistance and a fear of the treatment. In turn structural barriers apply to the organization of the therapy, infrastructure, personnel, and the therapeutic program. A comparison of barriers experienced by people with gambling disorders and substance use disorders showed that they are largely similar, but people with gambling disorders also experience specific barriers. Empirical studies focused specifically on treatment needs of people experiencing gambling disorders may improve an offer of help for them. More adequate treatment options could contribute to the increasing in the number of people who start the treatment. It can result in improving their quality of life and may have positive impact on public health.

## Introduction

Such factors as an increasing availability of gambling over the last decades, a low social knowledge on gambling disorders, and a perception of gambling rather in terms of moral weakness than a medical disorder have an impact on a social acceptance of gambling behaviors (Petry and Blanco 2013; St-Pierre et al. 2014; Hing et al. 2015).

A prevalence of gambling is diversified and depending on the region of the world. In the United States 70–90% of adult population has experience of gambling within a whole of life (Ladouceur et al. 1999; Raylu and Oei 2002). Studies conducted in different countries show that a prevalence of problem gambling in 12 moths before the study vary between 0.3% in Sweden and 5.3% in Hong Kong (Wardle et al. 2011). In Poland in the group of 15 years-old and more, in the period of 12 moths before the study, the prevalence of problem gambling was set at level of 0.7% (CBOS Report 2015).

Existing studies show that treatment is undertaken by 10–20% of people with gambling disorders (Volberg et al. 2006; Slutske et al. 2009). The majority of gamblers do not seek treatment (Cunningham 2005) Almost three quarters (71%) people with pathological gambling had never sought a professional treatment as well as an assistance in self-help groups (Suurvali et al. 2008). A study conducted in Australia among 2060 adults indicated that from 24% of problem gamblers declared a need of help, 17% accessed one or more services (Davidson and Rodgers 2010). People with gambling disorders whose problems are more severe, more often than others, decide to initiate a treatment (Pulford et al. 2009).

Reasons why people with gambling disorders do not initiate treatment are complex and include social, cultural, individual and structural factors. The studies usually show barriers to the treatment divided into two groups depending on their background: barriers arising from personal beliefs (individual barriers) and connected with the structure of the treatment (structural barriers). In the first group following barriers can be distinguished: denying that someone has a problem with gambling/non-acknowledgment of gambling problems, beliefs that someone can solve problem with gambling by themselves, unwillingness to receive an therapeutic advice related to the gambling and to talk about private life, beliefs that financial problems can be solved by gambling, a shame, a fear against stigma, a pride, a willingness to keep the problem with a gambling in a secret, a lack of support in the process of changing behavior, doubts about a quality and effectiveness of the treatment, feeling too overhelmed by other issues, not liking to be told what to do, rationalizing that gambling was permissible since the person had no other problematic behaviors as well as feeling of loneliness because of gambling disorders are rare among others treated. In the group of structural barriers respondents mentioned a lack of awareness of services, a large distance from the place of residence to the facility, high costs of the treatment, lack of culturally and linguistically appropriate services, opening hours of clinics not relevant to the needs, unsatisfying program rules including abstinence, a lack of information from therapists about treatment regiment, high availability of gambling (Evans and Delfabbro 2005; Gainsbury et al. 2014; Suurvali et al. 2009).

This article aimed at the presentation of barriers to the treatment for people with gambling disorders identified in discussed study in comparison to barriers experienced by people with alcohol and drug disorders based on the literature review.

Two main research questions were formulated:

1. What barriers to the treatment are identified by people with gambling disorders and professionals involved in helping them?
2. What are the differences and similarities between barriers to substance treatment and barriers to gambling treatment?

## Methodology

### Local Context of the Study

In Poland, as in other countries, therapeutic offer for people with gambling disorders is very often the same offer which is addressed to people with alcohol or drug disorder (Toneatto and Brennan 2002). There is a lack of facilities dedicated exclusively to people with gambling disorders. If gambling disorders comorbid with alcohol or drug dependence, a major concern is a disorder related to the substance. People with gambling disorders, similar to those with alcohol or drug disorders, can receive an inpatient or an outpatient form of treatment as well as nonmedical support—Gamblers Anonymous. Behavioral, cognitive and cognitive-behavioral approaches dominate. Availability of brief interventions and programs aimed at reducing of gambling is marginal (PARPA web page on alcohol treatment system 2016, PARPA web page on therapist certifying program 2016, KBPN web page on drug treatment system 2016, KBPN web page on system of therapist certification 2016).

### Selection of the Respondents: Sample Selection

Interviews were conducted with people with gambling disorders, social workers, therapists employed in addiction treatment facilities, General Practitioners and psychiatrists. Selection of the respondents was based on purposive sampling. The aim of such selection was to choose only those respondents who would provide complete and comprehensive information from the perspective of research questions (Wasilewska 2008).

Research sample consisted of 90 respondents and covered 30 interviews with people with gambling disorders and 15 interviews with each group of professionals—social workers, General Practitioners, psychiatrists and therapists. Inclusion criteria for professionals were status of employment in facility where treatment of gambling disorders is offered and their profession. In turn, inclusion criteria for people with gambling disorders was a diagnosis of gambling disorders confirmed by a psychiatrist. There were two kinds of places where respondents were enrolled—alcohol and drug treatment facilities and meetings of Gambling Anonymous (GA). In the case of the first place, patients were recruited by therapists and then contacted by researchers to conduct an interview. In the second case—the researcher contacted the leader of the group of Gambling Anonymous asking to provide information about the study on the meeting. Those interested in participating in the study contacted with the researcher directly and the interview was carried out at a convenient time and place.

### Characteristic of the Sample

The vast majority of the sample in the group of people with gambling disorders were male; the study included only three females with diagnosis of gambling disorders. Average age in this group was 38.3 years (SD = 10.827 years). The youngest respondent was 25 years-old and the oldest—63 years-old. Due to the fact that the study was conducted in Warsaw majority of respondents resided in this town, however about 25% (n = 8) lived outside of the city, commuting to the facility. More than half (60%, n = 18) of respondents had a university degree (bachelor or master degree). In the study, there were no people with primary and lower secondary education, only 10% (n = 3) had a vocational education. Almost everyone had a regular source of income, about 70% (n = 20) of the respondents were employed on tenure, almost quarter (n = 7) had his own business and the rest were retired. Only one person was unemployed.

The most popular gambling game (answers: often and very often) were slot machines (56.7%, n = 17), casinos (53.3%, n = 16) and online gambling (43.3%, n = 13). On the other hand, the least popular type of gambling (answer: I did not gambled) were horse race betting (76.7%, n = 23), SMS lotteries (73.3%, n = 22) and sport betting without using the internet (53.3%, n = 16).

The group of professionals was dominated by females, which constituted 70% (n = 42) of the sample. Average age was 42.9 years-old (SD = 12.012 years), and varied depending on the group. The highest average age was noted in the group of psychiatrists 44.4 years (SD = 11.115 years) and the lowest among therapists—40 years (SD = 11.473 years). Average age for General Practitioners was 43.7 years (SD = 13.767 years) and for social workers—42.9 years (SD = 12.264 years). The vast majority of the professionals lived in Warsaw, only a few resided outside the city.

### Research Tools

Three types of guidelines to conduct semi-structured interviews were developed—first one for people with gambling disorders, second—for social workers and the last one for professionals employed in the medical sector: General Practitioners, psychiatrists and therapists. Interview for people with gambling disorders was divided into six sections: experiences with treatment (the reason for entering treatment, the circumstances in which the respondent realized the problem, seek help outside the medical sector, reasons for choosing facility, difficulties in obtaining assistance), assessment of available treatment offer for people with gambling disorders (positive and negative experiences with treatment), social perception of people with gambling disorders based on the individual experiences, recommendations for improving treatment offer, types of preferred gambling games and comorbidity issue.

Second type of guideline was designed for social workers and included questions on reasons for seeking help in social welfare centers by people with gambling disorders, existing offer of social welfare for people with gambling disorders, demand for such offer among people with gambling disorders, perception of people with gambling disorders by social workers, influence of stigmatization on cooperation with people with gambling disorders, and recommendations how to improve offer of welfare centers.

Finally, guideline intended for therapists, General Practitioners and psychiatrists let to search reasons and circumstances of seeking treatment by people with gambling disorders, available offer of the assistance and treatment as well as patient’s experiences with seeking help outside the medical sector, stigmatization of people with gambling disorders and recommendations for improving situation in treatment.

All kinds of guidelines included section which allow to collect socio-demographic data such as age, place of residence, marital status, education level and employment.

### The Study Protocol and Data Analysis

Individual interviews were conducted in the first half of 2015. Before the proper phase of the study, the pilot interviews were carried out (two interviews with respondents from each sample group). After the pilot study, interview guidelines were revised. Interviews from the pilot phase were included to the study material.

The study was anonymous, opinion of the respondents were denoted only by a number; personal data were not collected. All respondents were informed about the aim of the study and signed the consent form for participation in the study. All interviews were recorded and then transcribed.

Each interview was analyzed separately. The analysis was initiated by reading full text and making notes on the margin of the interview. In the next step, relevant codes were made to cover topics interesting from the perspective of the aims of the study. Then, codes were aggregated into thematic categories which were assigned to the broader categories—dimensions. Coding and data analysis was made manually, without using any software.

Access to the treatment has been conceptualized in numerous ways (Levesque et al. 2013). Within health care, access is understood in terms of access to a service, a provider or an institution and defined as the opportunity or ease with which consumers are able to reach appropriate services in proportion to their needs (Whitehead 1992). While the access issue is often used in relation to factors determining the initial contact or use of services, opinions differ regarding aspects included within access and whether the emphasis should be put more on analyzing characteristics of the providers or the actual process of care (Frenk 1992).

The analysis in this study covered the factors that hinder the decision to undertake treatment or refrain from such a decision, as well as factors that appear already in the course of treatment. The latter factors can reduce the chances of keeping the patient in the treatment and influence the decision to take treatment again if need arises. In the first case answers concerned difficulties in obtaining assistance, in the second—negative experiences with treatment were analyzed.

Furthermore, the analysis takes into account dimension of the source of information, so statements of patients, social workers, General Practitioners, psychiatrists and therapists were encoded separately.

The authors had to make a decision regarding the presentation of research material in the article. It was possible to present the material with regard to several dimensions: to describe barriers from the perspective of people with gambling disorders and professionals, or/and present barriers divided into barriers to access to treatment and negative experiences at the stage of taking treatment. Another way was divide material into individual and structural dimensions. It was chosen by authors for two reasons. Firstly it was the way to avoid too many repetitions, as a lot of barriers mentioned by professionals overlapped with those mentioned by patients. Secondly this presentation facilitated comparison of barriers revealed in this study with barriers identified in other studies as it is the most widespread way of presenting barriers regarding the discussed issue.

It should be noted that the most barriers can be described with regard to both perspectives: individual and institutional. For example, little knowledge of existing therapeutic offer may have an individual dimension, if the person does not have the motivation to become familiar with the offer or institutional, when there is no easy access to the knowledge about treatment services. For this reason, the authors when deciding how to qualify particular barrier took into consideration the context in which the barrier was recalled. This distinction was largely a contractual nature.

### Review of the Articles About Barriers to the Alcohol and Drug Treatment

The aim of the review was to identify the publications related to barriers to the alcohol and drug treatment facilities. The articles to the review were identified in the Medline database with using search words *barriers* and *alcohol dependence* (162 articles), and search words *barriers* and *drug dependence* (142 articles). Based on abstracts we included 7 articles to the analysis, which directly corresponded to the aim of the study. The review also included articles identified in the references of the eligible publications (4 articles).

### Ethical Approval

Ethical approval to conduct the study was obtained from Bioethical Commission of the Institute of Psychiatry and Neurology from Warsaw, Poland (ref. 24/2015).

## Results

### Individual Barriers

Barriers in access to the treatment may result from the therapeutic requirements that are difficult to accept by potential patients. Many people with gambling disorders feel a fear and an aversion to speaking about their problems at the forum of therapeutic group. In the opinion of respondents, necessity of speaking about personal experiences, problems and feelings associated with gambling problems to the group of strangers, may result in failing to take the treatment. The necessity of admitting to yourself and your family, that you are an addict is inseparably linked to a sense of shame.

A shame accompanies every addiction. People drink for years, take drugs, gamble and shame appears in different situations. On the other hand, going to the treatment is associated with a completely different sense of shame. Because taking treatment is like admitting the problem, which for years they did not want to admit. Such a shame to himself, they conclude: Damn I’m a gambler, I have to go to the clinic. (TR2703_M_1^1^)

Beliefs that gambling is not a disease and it is impossible get addicted from it as well as possibility of self recovery in the situation of addiction are factors which influence a decision to start treatment or not.

At the meetings of Gambling Anonymous I found out that this disorder is a disease. Earlier I was convinced that something is wrong with me, that I’m stupid, and so on. (G1504_M_1)

The internal difficulty is that, as with any dependent person: “I can handle myself,” such conviction—“I can do it”, “it would be somehow”. Once, at the group meeting we identified three biggest scam of addicts. First, “somehow it will be”, second—“I can handle alone,” and third—“I will not drink/will not gamble/will not take drugs since tomorrow.” That whishes, which never come true—they have no chance for it, because therapy of dependence is based on the group meetings aimed at mutual support. (TR1902_K_1)

A lack of internal motivation to starting treatment can also be classified as a barrier. Usually people with gambling disorders do not see the need for a specialized treatment, they do not take it on its own initiative but rather under pressure of family or partner.

A person with gambling disorders is under external pressure, mostly family pressure (…) When the family realizes that the money disappear, and there are gambling debts, exerts pressure and this is one of factors that lead to treatment (PS2804_M_1)

An important barrier to the decision to undertake treatment is the fear of stigmatization. Apart from obtaining the label of addict person, the decision about treatment may involve the disclosure of various facts, such as robbing and cheating family, losing large amounts of money, which put a person with gambling disorders in a bad light. They can meet with condemnation and lack of confidence that does not disappear despite taking the treatment.

People do not trust us (people with gambling disorders—authors), and this is understandable. I also can not trust myself. No matter how long I do not play, I can never promise, that I will not play to the rest of my life. Recently a friend of my wife borrowed from her a little bit of money. Give this money back to me, she stressed that money should be returned to my wife personally. As if she warned me that I did not go to the casino. So it’s that kind of things. (H1504_M_2)

People with gambling disorders are often treated in groups dominated by people with substance disorders. As they are in a minority, thus they can feel isolated and misunderstood.

I was assigned to the group of people with alcohol and drug dependence or other psychoactive substances, and I felt a little bit lonely because there were only two more people with gambling disorders and at later stages of therapy I was alone. Topics discussed during the therapy were suitable for people with alcohol dependence and not for people with gambling disorders. (G2505_M_1)

Some forms of treatment and support are associated with a greater sense of stigma. Psychiatric treatment is one of examples. Treatment in the psychiatric sector is burdened with fear of stigma associated with the identification with mentally ill people, which seems to be perceived as more severe than stigma of addict. Another type of assistance charged with various stereotypes is a social welfare. Using a social welfare services can be associated with an admission of failure in a life. According to social beliefs an offer of this type of institution is addressed to people who are poorly integrated socially, shiftless, without any incomes, without prospects for the future.

### Structural Barriers

Most of barriers identified by respondents are related to the structure and organization of treatment and help. A large group of barriers concerns the quality of offer addressed to people with gambling disorders, including the treatment program, skills of therapists and therapeutic relationships. In the opinion of most patients and professionals, there is a lack of treatment offer for people with gambling disorders. Only few clinics have a therapeutic offer which is designed for people with gambling disorders. People searching for help are often guided to substance addiction treatment facilities but their offer is perceived as inadequate, does not fulfill the needs of people with gambling disorders as is concentrated on substance dependence issues.

If at the therapeutic groups patients are mixed, people with gambling disorders with people with alcohol dependence, we often talk only about alcohol or about drinking. And people with gambling disorders must translate the content of therapeutic program by themselves. (TR2603_K_2)

A small proportion of people with gambling disorders in the treatment group results in therapeutic program focused on alcohol or drug disorders. There is a lack of consideration of specific nature of gambling disorders. Educational materials are tailored mainly to the requirements of therapy of alcohol or drug disorders. All this makes that people with gambling problems have a sense of inadequacy of therapy and affect their maintenance in the therapy.

I think there should be different therapeutic tools for people with alcohol dependence and gambling disorders, for example those brochures which we get are mainly for people with alcohol dependence. People with gambling disorders are not able to translate the language which is addressed to alcohol dependence into their own dependence. They say “it is not for me because it does not concern me”. (G1004_K_2)

These therapeutic programs are inadequate, because many times we received materials from therapists which were designed for alcohol dependent people and we had to modify them by ourselves. Even it is not the point to changing the word from alcohol to gambling because there are some differences in these diseases. (G3003_M_2)

Some barriers are related to the nature of therapeutic relationship. It happens that the staff attitudes to patients are characterized by a lack of trust and by a desire to exercise personal control over the patient. This is reflected for example in the requirement of justifications and comprehensive explanation of absence from the therapy. Such procedures are difficult for respondents to accept.

I had to cancel one or two individual meetings with therapist because of situation at work, and so on. I just could not be at the meeting. Perhaps these is connected with a lack of confidence to patients, but I do not really love these questions Why? What happened? The lady in the registration does not have to know what is the reason of my absence at individual meeting (G3103_M_1)

Another allegation at therapists is their insufficient professional experience for conducting gambling therapy. In the opinion of people with gambling disorders, many have little or no at all experience in treatment of such a disorder. Some of them, treat people with gambling disorders, although they do not have substantial knowledge in that field, using experience in therapy of people with alcohol or drug dependence.

I saw that therapists are moving a little intuitive in these field and it is not supported by some experience and knowledge. (G3003_M_2)

In my case, the first therapist was an expert on alcohol dependence, so she told me all the time that this is exactly the same, but in some areas it is not the same. (G2505_M_1)

In the opinion of people with gambling disorders, a size of treatment groups can be a barrier which restrains potential patients from taking the therapy. Too many patients in one group makes contact with therapists difficult and hinders active participation in the therapy. The experience of patients shows that in such a case there is not enough time for training practical skills.

There is no time to do exercises, because there is so many people, so much expressions, so much you have to comment that just three hours is not enough. Therapeutic groups should be smaller. In general should be 8–10–12 people in the group, and in mine is 18. (G0806_M_1)

Another large group of barriers covers various aspects of availability of treatment. One of them is a waiting time for the treatment. Many of respondents claimed that long waiting time does not favor the perseverance in decision on starting the treatment.

I wanted to get help immediately. When the excitement wore off, I have started to looking for meetings of Gambling Anonymous, or anything to talk to someone because two weeks of waiting seemed an eternity. Two weeks it is fourteen days, when you are at very high emotion, you do not gamble but you want, it is hard to persist. Without meetings I would not be able to maintain the abstinence. (G1004_K_2)

People with gambling disorders stressed that hours of therapy are inappropriate for those working and it is a problem to reconcile all daily duties (for example work) with treatment regime.

You could go on the therapy to the day-care center, but it starts at 8 a.m. and finish at 4 p.m. every day and takes two month. It is possible to get sick-leave but two months is a quite long period of time and you can drop out from the labor market. I wonder what the employer would say. (G2605_M_1)

Another barrier is limited access to free treatment for people who do not have medical insurance. In the case of alcohol or drug users without insurance the cost of treatment reimburses the Ministry of Health. These regulations, however, do not include people with gambling disorders. Other problem is that some of addiction treatment facilities do not have contract for treating gambling disorders with National Health Fund. Of course there are private facilities at the medical services market, which offer a treatment for people with gambling disorders, but most of them can not afford that.

There is a group of patients who can not take the treatment—uninsured patients. We can not help them, because they are uninsured. When it comes to people dependent to alcohol or drugs—a person uninsured are entitled to receive free treatment of their dependence. This is financed from other sources, from Ministry of Health. There is no funds for treating people with gambling disorders who are uninsured and this is in fact big problem. This is a serious gap. (TR1902_K_1)

The barrier can be difficulty in meeting the requirements for participation in therapy. Some respondents claimed that therapy consumes so much time that they must give up other activities. Some people with gambling disorders do not take into account treatment in inpatient clinic because they have a feeling that they have to work to pay off their debts.

There are some inpatient clinics which also treat people with gambling disorders but no-one so far was interested in this offer. They just want to combine work with therapy because they have a feeling that they must immediately work off their debts, more than other people who also have debts which arose due to other kinds of dependence. (TR2603_K_2)

Some structural barriers are specific and related to the type of institution. In the case of primary health care, patients do not know if gambling disorders can be treated by General Practitioners. There is a widespread belief that primary care physicians treat only somatic disorders. In the psychiatric care sector, the most important barrier is a lack of knowledge that there is a possibility of a psychiatric consultation without a referral. The barrier for both, General Practitioners and psychiatrists may be their superficial knowledge of gambling disorders. Most barriers occurring in social welfare services are associated with bureaucratic requirements. Firstly, the problem gambling does not “exists” in the questionnaires or application forms and is not recognized in the diagnosis. In the effect, gambling disorders are disclosed by coincidence, for example when family report about it or in the case of co-ocurring disorders.

I think that during ten years of my employment in social welfare center maybe three or four times someone reported a problem of gambling. I think it is due to the specificity of the facility, because gambling is not included in the list of reasons that predispose to benefit from social support. So gambling is not included in the questionnaire of interview; in any internal diagnoses. It is really by chance only if social worker notices gambling disorders, because for example the family report a problem or because there was comorbid dependencies. (SW1103_M_1)

Secondly, the problem of gambling can not be disclosed in official documents relating to the patient, because there is a concern that granted funds can be wasted by allocation on gambling. In the effect, the legitimacy of provided services is undermined.

In every case we are looking for the causes of the financial troubles. We consider, why this family is in the difficult situation. In this particular situation, people have a reason to hide gambling, because regulations of the Act on Social Welfare says that waste of any resources causes a refusal of benefits. (SW1604_K_1)

Thirdly, the social welfare procedures impose on the beneficiary various commitments to meet. These procedures are barriers which refrain people with gambling disorders from looking for the support and seeking help in these institution.

It seems to me, that Social Welfare Center will be one of the last places where people with gambling disorders apply for help. Social Welfare Center is the institution of control, and is perceived in such a way. So they know very well, if they turn to us for help, they will be under supervision. The Center will be the last place where they come, because they have enough control, for example from their families. They do not contribute themselves another source of control. (SW1103_M_1)

### Barriers to the Treatment of Alcohol and Drug Disorders: Results of the Literature Review

#### Individual Barriers

A major individual barrier identified by people with alcohol and drug disorders is stigmatization. It is confirmed by epidemiological as well as qualitative studies that fear of being labeled an alcoholic or drug addict delays decision on seeking treatment (Digiusto and Treloar 2007; Radcliffe and Stevens 2008; Keyes et al. 2010; Wallhed Finn et al. 2014; Gilchrist et al. 2014; Wieczorek 2016). Drug users who want to start treatment may be accompanied by a fear of being reported to the police while they will register at the facility. It can be visible especially in those countries where possession of drug or drug use is penalized (Bobrova et al. 2006). In this case, fear concerns the institutionalization of stigmatization.

Other individual barrier which was identified by Swedish researchers in the group of people with alcohol disorders was limited knowledge about the consequences of heavy drinking on health, as well as about different kinds of interventions addressed for alcohol disorders. The knowledge of respondents about treatment was limited to involuntary pharmacological treatment (using Disulfiram), residential treatment and lifelong abstinence (Wallhed Finn et al. 2014).

In the study of cannabis users who did not seek the treatment, the main reason for that was belief that they are able to copy with the problem without help and that cannabis problem does not require professional intervention (van der Pol et al. 2013).

Other individual barriers include a lack of support from ‘significant others’ and low personal disposition—motivation and recognition of the problem attitudes (Gilchrist et al. 2014). It happens that patients deny that they have a problem of dependence (Laudet et al.,^2009^).

#### Structural Barriers

Structural barriers relate to the effectiveness and quality of treatment, including the therapeutic relationship. Indicators of low effectiveness are high failure rates, short remissions and then continuation of drug use or drinking. Negative opinion on the effectiveness of the treatment could lead to refraining from the therapy (Bobrova et al. 2006; Digiusto and Treloar 2007; van der Pol et al. 2013; Gilchrist et al. 2014). An important obstacle to the maintenance in the treatment may be unsatisfactory relationship with the therapists because of lack of trust and patient acceptance, lack of support from health professionals (Digiusto and Treloar 2007; Laudet et al. 2009; Gilchrist et al. 2014).

Some barriers relate to requirements that patients must meet to initiate the treatment or then during the therapy. Sometimes entry criteria may prevent the initiation of treatment, for example two weeks abstinence period (Digiusto and Treloar 2007; Gilchrist et al. 2014). The necessity of obtaining a referral from General Practitioner to begin a therapy can be perceived as a barrier, because people with alcohol and drug disorders do not wish to admit about their problem (Welbel et al. 2013; Gilchrist et al. 2014). The studies also highlight difficulties in financing therapy. Financial difficulties identified by Russian and Australian drug users refer to high costs of treatment (Bobrova et al. 2006; Digiusto and Treloar 2007). An abstinence for the rest of life as a treatment goal is not acceptable for many people. Respondents do not want to give up use drugs or drinking alcohol and maintain total abstinence for the rest of their life. They were more open to cutting down or learn drinking in a controlled manner rather than choosing total abstinence (Laudet et al. 2009; Wallhed Finn et al. 2014).

Research conducted by Laudet et al. (2009) aimed at identification of reasons for leaving treatment from the perspective of outpatient treatment clients. These reasons can be considered as barriers to treatment. The study confirmed that program attendance interferes with other important activities (e.g. work, school) what influence on treatment compliance.

Another group of issues concerns the availability of treatment. For people with alcohol and drug disorders a long distance to drug and alcohol treatment facilities constituted a barrier. Gathering to the facility is time consuming and costly, especially in the rural communities (Digiusto and Treloar 2007; Gilchrist et al. 2014; Wieczorek 2016). Australian and US surveys conducting in the group of illicit drugs users show that the most common barrier was long waiting time for the treatment and lack of places available in the facility (Redko et al. 2006; Digiusto and Treloar 2007). Other barrier is limited opening hours of facilities, e.g. at the weekends (Welbel et al. 2013).

## Discussion

The aim of the study was identification of barriers to the treatment for people with gambling disorders revealed in the described study comparing with barriers experienced by people with alcohol and drug disorders. Individual barriers in the perception of people with gambling disorders cover fear and aversion to speaking about their problems at the forum of therapeutic group, the difficulty to recognize that you are a dependent person and admitting that before the family and others. Most of the people with gambling disorders do not see the need of starting the treatment, because they are convinced that treatment is intended for people with alcohol or drug disorders. Furthermore they believe in the possibility of self recovery. Treatment in psychiatric facilities and being a client of social welfare sector can be associated with a greater feeling of stigma in comparison with other forms of help or assistance.

There are similarities between the individual barriers highlighted by persons with gambling and alcohol or drug disorders. In a study of cannabis users appears the belief in the opportunity to deal with the problem without professional help (van der Pol et al. 2013). People with gambling disorders are often convinced that gambling is not a disease and that self recovery is possible in the situation of addiction.

Among people with drug and alcohol disorders, similarly like in the group of people with gambling disorders, stigmatization was seen as an obstacle to the treatment. Stigma and shame are barriers that seem to be the most frequently identified in both groups: the substance-dependent and gambling (Digiusto and Treloar 2007; Radcliffe and Stevens 2008; Keyes et al. 2010; Wallhed Finn et al. 2014; Gilchrist et al. 2014; Wieczorek 2016, Evans and Delfabbro 2005; Gainsbury et al. 2014; Suurvali et al. 2009). But among people with gambling disorders it is not seem to be so important as in the group of people with alcohol and drug dependence. The reported study found that exposure to condemnation in the case of gambling can be much weaker than in the case of alcohol or drug disorders, because it is a relatively new phenomenon, socially not defined as dependency.

The vast majority of barriers identified in the study have administrative and organizational background. First of all, there is a lack of offer addressed exclusively to the people with gambling disorders, thus the offer is perceived as inadequate to the needs. This means in the practice that examples which are discussed during the therapy are not related to the everyday life of people with gambling disorders and educational materials do not address their specific needs. As a consequence, patients do not engage enough in the treatment, do not identify themselves with the group and they have a feeling of marginalization during the therapy. For people with gambling disorders treatment in the same therapeutic group with people with alcohol or drug disorders who constitute the majority in the group, is a barrier which discourage them to start the treatment and decrease the satisfaction with treatment.

A whole group of barriers is related to the patient-therapist relationship and competences of therapists and other staff. Respondents claimed that therapists have not enough experience in the treatment of gambling disorders, they provide the therapy despite they do not have theoretical background. Personnel tends to overcontrol patients, e.g. comprehensive explanation absence from therapy is required, which puts patients in the role of person which do not deserve to the trust.

Other barriers identified by respondents refers to the long waiting time for the treatment, limited possibilities of free treatment (people with gambling disorders without insurance cannot take the therapy), difficulties in reconciliation of the therapy and the employment. Some of the barriers are related to the particular institutions. In the case of the primary health care as well as in the psychiatric care, patients do not know that they can ask for help General Practitioners or visit a psychiatrist without referral. In terms of social assistance, the barrier is that gambling disorders are not included in administrative forms as a problem which entitle to getting support. If gambling disorders are exposed in official client documentation it could be a concern that financial support will be wasted. Otherwise clients have to fulfill the contract for the provision of support. The progress in implementing the contract is evaluated by a social worker, which can be perceived as a form of control.

Discussed structural barriers are in many areas similar with those which are identified by people with alcohol or drug dependence, e.g. access to cheap or free treatment (Bobrova et al. 2006; Digiusto and Treloar 2007), waiting time for the treatment (Redko et al. 2006; Digiusto and Treloar 2007), therapy in unfavorable hours (Welbel et al. 2013), inability to reconcile the treatment with other obligations (Laudet et al. 2009), attitudes of therapists (acceptance for patient, trust) (Digiusto and Treloar 2007; Laudet et al. 2009; Gilchrist et al. 2014).

Still, there are other barriers that are not found out in this study, but emerge in the other studies searching for barriers faced by people with gambling disorder. The study of Evans and Delfabbro (2005) revealed that people with gambling disorders are convinced that gambling is an attractive way to spend time and entertainment, and they reluctant to give it up. Doubts efficacy of the treatment were expressed by respondents of one of the study reviewed by Suurvali et al. (2009). Australia Productivity Commission (1999) study shows that an important factor that influences satisfaction with the proposed offer is the geographical distribution of services and their hours of operation.

Interestingly, the respondents of discussed study not treated geographic localization of facility as a barrier, although they claimed that there is a low number of facilities providing treatment for gamblers. One explanation is that the study were conducted in Warsaw where the public transport is pretty good. It can be that people with gambling disorders believe that their disorder is so rare that they do not even expect a network of institutions dealing with this problem. Another explanation is that patients avoid treatment centers, which are located close to their place of residence because they do not want to meet friends or neighbors (Wieczorek 2016).

Novelty of this study is to identify barriers which relate to the specific forms of assistance and treatment offered to people with gambling disorders. Especially interesting is distinction between the nature and severity of the stigma depending on the type of offered help. In the psychiatric sector greater fear against stigma is connected with the risk of identification with mentally ill people. Another type of an assistance charged with various stereotypes is a social welfare. Using this type of services involves not only the stigma of the addict person but additionally stigma of life loser.

This study allowed for recognizing the barriers to treatment which are common to people with substance and gambling disorders, but also specific to those with gambling disorders. It is important to capture the specificity of gambling clients, who are often treated in the system of help offered to people with alcohol and drug disorders.

Moreover, this study enables to recognize the barriers to gambling treatment both at the individual and structural level from the perspective of clients of services offered gambling treatment and the various professional groups involved in helping them. Criticism regarding studies of barriers to treatment is related to using a self-reported information on barriers to seeking help and in particular information spontaneously expressed by respondents. Doubts concern to self—awareness of barriers and the reluctance to share with the researcher intimate thoughts or experiences (Suurvali et al. 2009). In the case of this study, the use of complementary source of knowledge (professionals) allowed least partially to respond to this problem. Professionals turned their attention to structural barriers that may be overlooked by patients.

To identify barriers in access to the treatment a qualitative approach was used, what allowed for taking into consideration opinion of the respondents expressed spontaneously. In quantitative research, respondents choose from a closed-ended barriers list that makes difficult or even impossible to identify new and non-obvious barriers. As Suurvali et al. (2009) stated open-questions can contain a richness of details and nuances difficult to replicate in closed-ended questions. Understanding the barriers, both at the objective and subjective level allows for a better understanding of what could contribute to increasing the availability and attractiveness of treatment.

And last, but not least studies on the treatment of gambling disorders are very scarce in Poland, and there is no studies investigating problem of barriers to the treatment. Meantime the results of this study may have practical application in improving the availability and quality of the treatment.

Some of barriers identified in current study may be due to the specifics of how treatment of gambling disorders is organized in Poland, e.g. in other countries access to specialized gambling treatment can be greater. In Poland problem of gambling is poorly recognized socially, and it is not always perceived in terms of addiction. Beliefs of respondents that gambling is not a disease and it is impossible get addicted from it influence the decision to start treatment. While in Poland, there are two government agencies that deal with the problem of alcohol and drugs disorders, the problem of gambling is rather neglected and is given much less weight in discussions on public health. This can results in insufficient professional background of therapists for conducting gambling therapy, because there is no system of certification of therapists who specialize in gambling disorders, as is in the case of alcohol and drug therapists. Another problem that can be attributed to polish treatment system is the mismatch between the content of the therapeutic programs and needs of people with gambling disorders.

The study has a number of limitations. In the study participated only those who already had experiences with gambling treatment, so identified barriers not include perspective of people who are outside the care system. The recruiting procedure of people with gambling disorders could have impact on answers of respondents as some of them were recruited via therapists.

## Conclusions and Implications

Studies focused on issues related to the access to the treatment for people with gambling disorders allow better understand needs of this group and improve an offer of help for them. Better meet the needs of people with gambling disorders could contribute to the increase in the number of people who initiate the treatment and maintain in the therapy.

Results of this study indicate the need for education of society about gambling as behavior from which one can become addicted and also about the various treatment options. Efforts are necessary to better respond to the treatment needs of people with gambling disorders and improve quality of treatment. These efforts should primarily include better professional preparation of therapists and other professionals to help people with gambling disorders, taking into account the problem of gambling disorders in the diagnosis, better fit the contents of therapeutic programs to the specificity of gambling disorders, creation of materials used in therapy tailored to needs of patient with gambling disorders and as far as possible establishing a therapeutic groups, which would be at least a couple of patients with gambling disorders.

Psychological barriers emerge in the studies as the most substantial (Hodgins and el-Guebaly 2000; Pulford et al. 2009). It is precisely these barriers are more difficult to overcome in comparison to structural barriers (Gainsbury et al. 2014). One of the major barriers are the shame and fear of stigmatization (Hodgins and el-Guebaly 2000; Pulford et al. 2009; Suurvali et al. 2009.) Web-based counseling has the potential to address these barriers (Rodda et al. 2013). Therefore creating a support network on the Internet should be considered, for people who for various reasons do not want to have contact with the health care services.

## Footnotes

1 The method of coding: TR—therapist; G—people with gambling disorders; GP—general practitioner; PS—psychiatrist; S—social workers; XXXX—number of the interview; M—male, K—female; Y—number of the interview conducted on the same day.

## References

[CR1] Bobrova N, Rhodes T, Power R, Alcorn R, Neifeld E, Krasiukov N (2006). Barriers to accessing drug treatment in Russia: a qualitative study among injecting drug users in two cities. Drug and Alcohol Dependence.

[CR2] CBOS Report (2015). Oszacowanie rozpowszechnienia wybranych uzależnień behawioralnych oraz analiza korelacji pomiędzy występowaniem uzależnień behawioralnych a używaniem substancji psychoaktywnych.

[CR3] Cunningham J (2005). Little use of treatment among problem gamblers. Psychiatric Services.

[CR4] Davidson T, Rodgers B (2010). 2009 Survey of the nature and extent of gambling, and problem gambling, in the Australian Capital Territory.

[CR5] Digiusto E, Treloar C (2007). Equity of access to treatment, and barriers to treatment for illicit drug use in Australia. Addiction.

[CR6] Evans L, Delfabbro PH (2005). Motivators for change and barriers to help-seeking. Journal of Gambling Studies.

[CR7] Frenk J, White KL, Frenk J, Ordonez C, Paganini JM, Starfield B (1992). The concept and measurement of accessibility. Health services research: An anthology.

[CR8] Gainsbury S, Hing N, Suhonen N (2014). Professional help-seeking for gambling problems: Awareness, barriers and motivators for treatment. Journal of Gambling Studies.

[CR9] Gilchrist G, Moskalewicz J, Nutt R, Love J, Germeni E, Valkova I (2014). Understanding access to substance treatment services in Europe: A multi-country users’ perspective. Drugs: Education Prevention and Policy.

[CR10] Hing, N., Russell, A., Gainsbury, S., & Nuske, E. (2015). The public stigma of problem gambling: Its nature and relative intensity compared to other health conditions. *Journal of Gambling Studies*, published online 20 October, 1–18. 10.1007/s10899-015-9580-8 PMC4993796 26487344

[CR11] Hodgins D, el-Guebaly N (2000). Natural and treatment-assisted recovery from gambling problems: A comparison of resolved and active gamblers. Addiction.

[CR12] KBPN web page on drug treatment system, available on 15.01.2016. http://www.kbpn.gov.pl/portal?id=106349.

[CR13] KBPN web page on system of therapist certification, available on 15.01.2016. http://www.kbpn.gov.pl/portal?id=104958.

[CR14] Keyes KM, Hatzenbuechler ML, McLaughlin KA, Link B, Olfson M, Grant BF (2010). Stigma and treatment for alcohol disorders in the United States. Amerciacn Journal of Epidemiology.

[CR15] Ladouceur R, Jacques C, Ferland F, Giroux I (1999). Prevalence of problem gambling: A replication study 7 years later. Canadian Journal of Psychiatry.

[CR16] Laudet A, Stanick V, Sands S (2009). What could the program have done differently? A qualitative examination of reasons for leaving outpatient treatment. Journal of Substance Abuse Treatment.

[CR39] Levesque J-F, Harris MF, Russell G (2013). Patient-centred access to health care: Conceptualising access at the interface of health systems and populations. International Journal for Equity in Health.

[CR17] PARPA web page on alcohol treatment system, available on 15.01.2016. http://www.parpa.pl/index.php/lecznictwo-odwykowe/system-lecznictwa-odwykowego.

[CR18] PARPA web page on therapist certifying program, available on 15.01.2016. http://www.parpa.pl/index.php/certyfikacja-terapeutow/program-certyfikacji.

[CR40] Petry NM, Blanco C (2013). National gambling experiences in the United States: Will history repeat itself?. Addiction.

[CR20] Productivity Commission (1999). Australia’s gambling industries.

[CR21] Pulford J, Bellringer M, Abbott M, Clarke D, Hodgins D, Williams J (2009). Reasons for seeking help for a gambling problem: the experiences of gamblers who have sought specialist assistance and the perceptions of those who have not. Journal of Gambling Studies.

[CR22] Radcliffe P, Stevens A (2008). Are drug treatment services only for “thieving junkie scumbags”? drug users and the management of stigmatized identities. Social Science and Medicine.

[CR23] Raylu N, Oei TP (2002). Pathological gambling. A comprehensive review. Clinical Psychology Review.

[CR24] Redko C, Rapp R, Carlson R (2006). Waiting time as a barrier to treatment entry: Perceptions of substance users. Journal of Drug Issues.

[CR25] Rodda, S., Lubman, D. I., Dowling, N. A., Bough, A., & Jackson A. C. (2013). Web-based counseling for problem gambling: exploring motivations and recommendations. *Journal of Medical Internet Research*, *15*(5), e99. Published online 2013 May 24. doi: 10.2196/jmir.2474. 10.2196/jmir.2474 PMC3668618 23709155

[CR26] Slutske WS, Blaszczynski A, Martin NG (2009). Sex differences in the rates of recovery, treatment-seeking, and natural recovery in pathological gambling: results from an Australian community-based twin survey. Twin Research & Human Genetics.

[CR27] St-Pierre R, Walker D, Derevensky J, Gupta R (2014). How availability and accessibility of gambling venues influence problem gambling: A review of the literature. Gaming Law Review & Economics.

[CR28] Suurvali H, Hodgins D, Toneatto T, Cunningham J (2008). Treatment seeking among Ontario problem gamblers: results of a population survey. Psychiatric Services.

[CR29] Suurvali H, Cordingley J, Hodgins DC, Cunningham J (2009). Barriers to seeking help for gambling problems: A review of the empirical literature. Journal of Gambling Studies.

[CR30] Toneatto T, Brennan J (2002). Pathological gambling in treatment-seeking substance abusers. Addictive Behaviors.

[CR31] van der Pol P, Liebregts N, de Graaf R, Korf D, van den Brink W, van Laar M (2013). Facilitators and barriers in treatment seeking for cannabis dependence. Drug and Alcohol Dependence.

[CR32] Volberg, R. A., Nysse-Carris, K. L., & Gerstein, D. R. (2006). California problem gambling prevalence survey. Final Report. Available on 12.01.2016. http://www.standupca.org/reports/CA_Problem_Gambling_Prevalence_Survey-Final_Report.pdf.

[CR33] Wallhed Finn S, Bakshi AS, Andreasson S (2014). Alcohol consumption, dependence, and treatment barriers: perceptions among nontreatment seekers with alcohol dependence. Substance Use and Misuse.

[CR34] Wardle, H., Moody, A., Spence, S., Orford, J., Volberg, R., Jotangia, D., et al. (2011). British gambling prevalence survey 2007. Executive summary. Available on 12.01.2016. http://www.natcen.ac.uk/our-research/research/british-gambling-prevalence-survey/.

[CR35] Wasilewska E (2008). Statystyka opisowa nie tylko dla socjologów.

[CR36] Welbel M, Matanov A, Moskalewicz J, Barros H, Canavan R, Gabor E (2013). Addiction treatment in deprived urban areas in EU countries: Accessibility of care for people from socially marginalized groups. Drugs: education, prevention and policy.

[CR37] Whitehead M (1992). The concepts and principles of equity and health. International Journal of Health Services.

[CR38] Wieczorek, Ł. (2016). Barriers in the access to alcohol treatment in outpatient clinics in urban and rural community. *Psychiatria Polska*, in press. 10.12740/PP/60345 28455900

